# Thrombin Inhibition Prevents Endothelial Dysfunction and Reverses 20-HETE Overproduction without Affecting Blood Pressure in Angiotensin II-Induced Hypertension in Mice

**DOI:** 10.3390/ijms22168664

**Published:** 2021-08-12

**Authors:** Agnieszka Kij, Anna Bar, Kamil Przyborowski, Bartosz Proniewski, Lukasz Mateuszuk, Agnieszka Jasztal, Anna Kieronska-Rudek, Brygida Marczyk, Karolina Matyjaszczyk-Gwarda, Anna Tworzydlo, Camilla Enggaard, Pernille B. Lærkegaard Hansen, Boye Jensen, Maria Walczak, Stefan Chlopicki

**Affiliations:** 1 Jagiellonian Centre for Experimental Therapeutics (JCET), Jagiellonian University, Bobrzynskiego 14, 30-348 Krakow, Poland; agnieszka.kij@jcet.eu (A.K.); anna.bar@jcet.eu (A.B.); kamil.przyborowski@jcet.eu (K.P.); bartosz.proniewski@jcet.eu (B.P.); lukasz.mateuszuk@jcet.eu (L.M.); agnieszka.jasztal@jcet.eu (A.J.); anna.kieronska@jcet.eu (A.K.-R.); brygida.marczyk@jcet.eu (B.M.); karolina.matyjaszczyk@jcet.eu (K.M.-G.); anna.tworzydlo@jcet.eu (A.T.); maria.walczak@jcet.eu (M.W.); 2 Department of Cardiovascular and Renal Research, University of Southern Denmark, J.B. Winsløws Vej 21, 5000 Odense, Denmark; cenggaard@health.sdu.dk (C.E.); pbhansen@health.sdu.dk (P.B.L.H.); BLJensen@health.sdu.dk (B.J.); 3 Chair and Department of Toxicology, Jagiellonian University Medical College, Medyczna 9, 30-688 Krakow, Poland; 4 Chair of Pharmacology, Jagiellonian University Medical College, Grzegorzecka 16, 31-531 Krakow, Poland

**Keywords:** 20-HETE, angiotensin II, endothelial function, MRI, nitric oxide, NO, thrombin activity, dabigatran

## Abstract

Angiotensin II (Ang II) induces hypertension and endothelial dysfunction, but the involvement of thrombin in these responses is not clear. Here, we assessed the effects of the inhibition of thrombin activity by dabigatran on Ang II-induced hypertension and endothelial dysfunction in mice with a particular focus on NO- and 20-HETE-dependent pathways. As expected, dabigatran administration significantly delayed thrombin generation (CAT assay) in Ang II-treated hypertensive mice, and interestingly, it prevented endothelial dysfunction development, but it did not affect elevated blood pressure nor excessive aortic wall thickening. Dabigatran’s effects on endothelial function in Ang II-treated mice were evidenced by improved NO-dependent relaxation in the aorta in response to acetylcholine in vivo (MRI measurements) and increased systemic NO bioavailability (NO_2_^−^ quantification) with a concomitant increased ex vivo production of endothelium-derived NO (EPR analysis). Dabigatran treatment also contributed to the reduction in the endothelial expression of pro-inflammatory vWF and ICAM-1. Interestingly, the fall in systemic NO bioavailability in Ang II-treated mice was associated with increased 20-HETE concentration in plasma (UPLC-MS/MS analysis), which was normalised by dabigatran treatment. Taking together, the inhibition of thrombin activity in Ang II-induced hypertension in mice improves the NO-dependent function of vascular endothelium and normalises the 20-HETE-depedent pathway without affecting the blood pressure and vascular remodelling.

## 1. Introduction

The endothelium constitutes a monolayer of endothelial cells (ECs) lining the inner surface of all blood vessels and is responsible for regulating the vascular tone and permeability, smooth muscle cell proliferation, blood cells adhesion, thrombotic processes, and vascular inflammation [1,2]. A disturbance of vascular homeostasis leads to the development of endothelial dysfunction defined as a reduction in nitric oxide (NO)-dependent vessel function [3]. The impairment of endothelial function can be a cause or a consequence of many cardiovascular diseases, including hypertension [4,5], stroke, and myocardial infarction [6]. 

The pathophysiology of hypertension is multifactorial and depends on the interplay between vascular, nervous, and immune systems [5,7], with a particularly important role being played by the renin–angiotensin system (RAS), which drives many of the consequences of hypertension as evidenced by the therapeutic efficacy of RAS inhibitors. 

The overactivation of RAS in hypertension is associated with the excessive generation of arachidonic acid-derived 20-hydroxyeicosatetraenoic acid (20-HETE), a strong vasoconstrictor, which potentiates systemic vascular bed responses to angiotensin II (Ang II), and additionally impairs endothelial function [8,9]. Impairment of endothelial function is often associated with a reduction in the biosynthesis of vasodilatory epoxyeicosatrienoic acids (e.g., 14,15-EET) identified as an endothelium-derived hyperpolarising factor [10].

In recent studies, the involvement of thrombin-dependent mechanisms in the development of endothelial dysfunction in hypertension [11] or diabetes [12] has been proposed. Apart from the pivotal role of thrombin in blood coagulation, thrombin also acts through protease-activated receptors (PARs) [13] expressed on endothelial cells [14], and their overactivation could result in the impairment of the endothelium barrier and activation of its pro-inflammatory and pro-thrombotic phenotype [15]. Moreover, thrombin-activated endothelial cells promote the adhesion of leukocytes to the vascular wall, leading to the overproduction and overexpression of pro-inflammatory selectins, adhesion molecules (e.g., ICAM-1, VCAM-1), or cytokines [16], further exacerbating endothelial dysfunction. Although the involvement of factor XI (FXI) and subsequent thrombin activation in the development of angiotensin II-induced vascular inflammation has recently been proposed [11], it is not clear whether thrombin inhibition results in the modulation of NO- and 20-HETE–dependent pathways and whether dabigatran affect Ang II-induced hypertension and endothelial dysfunction.

Accordingly, in the present work, we assessed the effects of the direct inhibition of thrombin activity by dabigatran on endothelial function in hypertensive mice after short-term (one-week-long) and prolonged (two-week-long) administration of Ang II. Our results demonstrated for the first time that dabigatran inhibited the development of endothelial dysfunction detected in vivo by magnetic resonance imaging (MRI), increased systemic NO bioavailability, normalised plasma 20-HETE concentration, and limited endothelial inflammation but did not lower elevated blood pressure and aortic thickening, all of which were induced by Ang II in mice.

## 2. Results

### 2.1. Effects of Dabigatran on Elevated Blood Pressure and Vascular Remodelling in Ang II-Induced Hypertension

**Figure 1:**
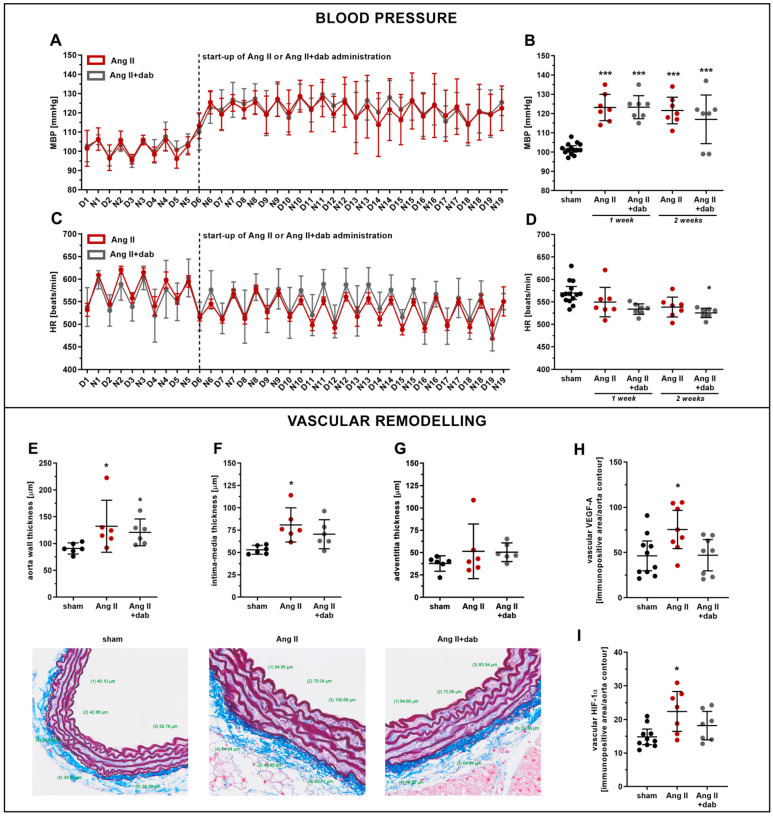
Effect of dabigatran on blood pressure, heart rate, and aortic remodelling in Ang II hypertensive mice. Mean blood pressure MAP (**A**,**B**; *n* = 7–14) and heart rate HR (**C**,**D**; *n* = 7–14) were continuously monitored by telemetry in mice subjected to *i.v.* continuous infusion of Ang II (144 µg/kg b.w per day; 2 weeks) via catheters. Quantitative analysis of aortic remodelling (OMSB staining) based on the thickness of the aorta wall (**E**; *n* = 6), intima-media (**F**; *n* = 6), and adventitia (**G**; *n* = 6) was performed in mice subjected to *s.c* administration of Ang II (1 mg/kg b.w per day; 1 week) via micro-osmotic pumps as well as vascular expression of VEGF-A (**H**; *n* = 8–10) and HIF-1α (**I**; *n* = 8–10) (IHC analysis). Data are shown as means (–) ± 95% CI (**I**) and considered statistically significant at * *p* ≤ 0.05 and *** *p* ≤ 0.001 using Tukey’s post hoc (**B**,**D**,**F**,**H**,**I**) and Kruskal–Wallis (**E**,**G**) statistical tests. * indicates statistical difference between sham mice and Ang II- or Ang II+ dab-treated animals. D—measurements during the day; N—measurement during the night.

The administration of Ang II to C57Bl/6J mice resulted in the elevation of mean blood pressure (MBP) (Figure 1A,B) and a slight decrease in heart rate (HR) (Figure 1C,D). The hypertensive effect of Ang II was already present 24 h after the initiation of *i.v.* Ang II administration and was sustained approximately at the same level throughout the two-week period of Ang II infusion (Figure 1A,B). The inhibition of thrombin activity by dabigatran did not lower Ang II-induced hypertension in mice as evidenced by telemetric blood pressure measurement over the two-week period (Figure 1A–D). 

Ang II-induced hypertension was associated with the vascular remodelling reflected by increased aortic wall and intima-media thickness quantified by combined orcein and martius scarlet blue (OMSB) staining of the aorta cross-sections (Figure 1E–G). Ang II-induced vascular wall remodelling was associated with an increased expression of vascular endothelial growth factor (VEGF-A; Figure 1H), hypoxia-inducible factor-1α (HIF-1α; Figure 1I) as well as stromal cell-derived factor-1α (SDF-1α; Figure S1D). However, dabigatran neither inhibited the vascular remodelling nor the expression of VEGF-A (Figure 1H), HIF-1α (Figure 1I), and SDF-1α (Figure S1D) induced by Ang II.

**Figure 2:**
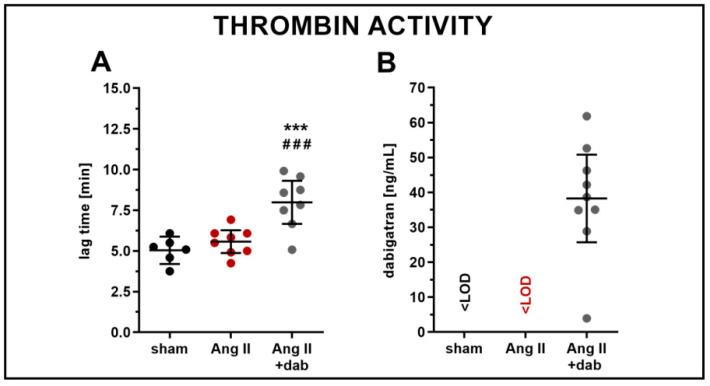
Effect of dabigatran on thrombin activity and its concentration in plasma in Ang II hypertensive mice. Thrombin activity expressed as lag time parameter (**A**; *n* = 6–8) and concentration of dabigatran in murine plasma (**B**; *n* = 9) were assessed in mice subjected to *s.c.* administration of Ang II (1 mg/kg b.w per day; 1 week) via micro-osmotic pumps. Data are shown as means (–) ± 95% CI (I) and considered statistically significant at *** *p* ≤ 0.001 and ### *p* ≤ 0.001 using Tukey’s post hoc test (**A**). * indicates statistical difference between sham mice and Ang II- or Ang II+ dab-treated animals; # indicates statistical difference between Ang II- and Ang II+ dab-treated mice.

Dabigatran administered to mice with a chow at a dose of 100 mg/kg b.w. per day effectively inhibited the thrombin activity as evidenced by the prolonged lag time (Figure 2A) and resulted in an average concentration of dabigatran in murine plasma of 38.26 ng/mL (Figure 2B). 

### 2.2. Effects of Dabigatran on Endothelial Dysfunction, NO- and 20-HETE-Dependent Function in Ang II-Induced Hypertension

**Figure 3:**
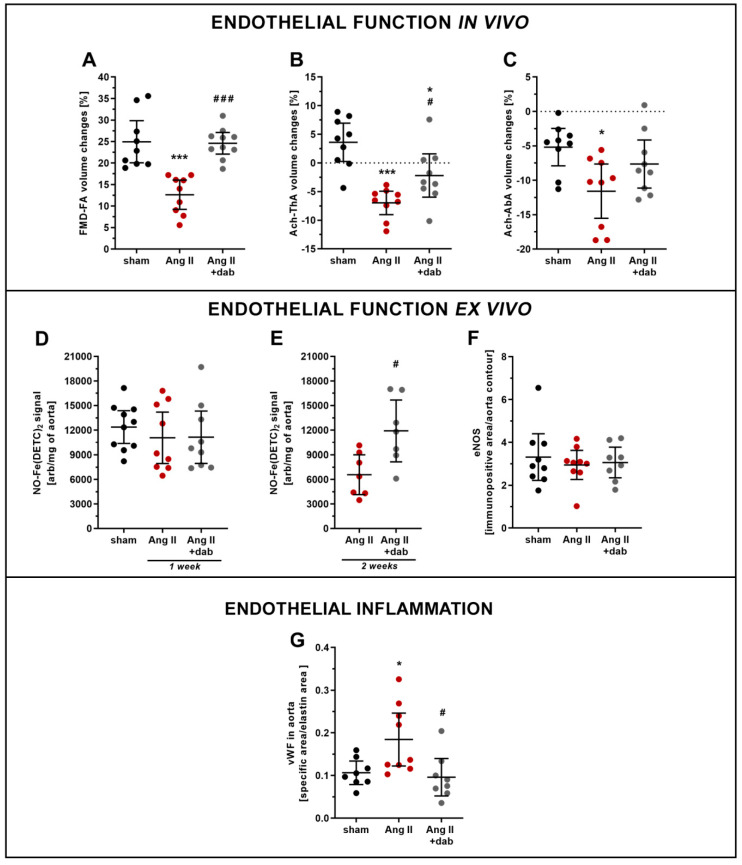
Effect of dabigatran on endothelial function and inflammation in Ang II hypertensive mice. Endothelial function assessed by MRI technique in vivo as changes in flow-mediated vasodilation response in the femoral artery FMD-FA (**A**; *n* = 9), Ach-induced vasodilation in thoracic aorta Ach-ThA (**B**; *n* = 9), and abdominal aorta Ach-AbA (**C**; *n* = 9) were evaluated in mice subjected to *s.c.* administration of Ang II (1 mg/kg b.w. per day; 1 week) via micro-osmotic pumps. Endothelial function assessed ex vivo based on the NO-Fe(DETC)_2_ signal measured by EPR as well as eNOS expression in aorta (IHC analysis) were evaluated in mice subjected to *s.c.* administration of Ang II (1 mg/kg b.w. per day; 1 week) via micro-osmotic pumps (**D**,**F**; *n* = 8–10) and *i.v.* continuous infusion of Ang II (144 µg/kg b.w. per day; 2 weeks) via catheters (**E**; *n* = 7). The aorta area positively stained for pro-inflammatory marker such as vWF (**G**; *n* = 7–9) was evaluated in mice subjected to *s.c.* administration of Ang II (1 mg/kg b.w. per day; 1 week) via micro-osmotic pumps. Data are shown as means (–) ± 95% CI (I) and considered statistically significant at * *p* ≤ 0.05, *** *p* ≤ 0.001, # *p* ≤ 0.05 and ### *p* ≤ 0.001 using Tukey’s post hoc (**A**–**D**,**F**,**G**) and *t*-test (**E**) statistical tests. * indicates statistical difference between sham mice and Ang II- or Ang II+ dab-treated animals, # indicates statistical difference between Ang II- and Ang II+ dab-treated mice.

Mice subjected to subcutaneous administration of Ang II (1 mg/kg b.w. per day) for one week displayed impaired endothelium-dependent vasodilation as evidenced by in vivo MRI-based measurements. Flow-mediated vasodilation response in the femoral artery (FMD-FA) as well as Ach-induced vasodilation in the thoracic (Ach-ThA) and abdominal (Ach-AbA) aorta were all diminished in Ang II-treated mice as compared with control mice (Figure 3A–C). Treatment with dabigatran profoundly attenuated endothelial dysfunction in Ang II-treated mice as evidenced by improved FMD-FA and Ach-ThA vasodilatory responses in vivo (Figure 3A,B). In contrast to in vivo MRI-based measurements, ex vivo production of NO in the aorta measured as NO-Fe(DETC)_2_ adduct (Figure 3D) as well as endothelial nitric oxide synthase (eNOS) expression in the aorta (Figure 3F) were not markedly affected by Ang II, irrespective of whether or not animals were treated with dabigatran. 

The development of endothelial dysfunction in Ang II-treated mice was associated with endothelial inflammation as evidenced by an increased endothelial expression of vWF, whereas concomitant administration of dabigatran prevented the increase in vWF expression (Figure 3G). 

The improvement of endothelium-dependent vasodilation by dabigatran was not associated with changes in the eicosanoid profile released by the AbA stimulated with arachidonic acid (AA, 1 µM). Neither Ang II administration nor Ang II with concomitant treatment with dabigatran significantly affected the biosynthesis of hydroxyeicosatetraenoic acids (HETEs) and epoxyeicosatrienoic acids (EETs) by the mouse aorta (Figure S2). Moreover, eicosanoid production in full blood using an ex vivo full blood assay did not reveal any notable changes in plasma eicosanoid profile and soluble hydrolase activity (sEH) expressed as EETs/DHETs ratio in Ang II hypertensive mice with or without dabigatran treatment (Table 1).

### 2.3. Effects of Dabigatran on Systemic NO Bioavailability and Plasma Concentration of 20-HETE in Ang II-Induced Hypertension

**Figure 4:**
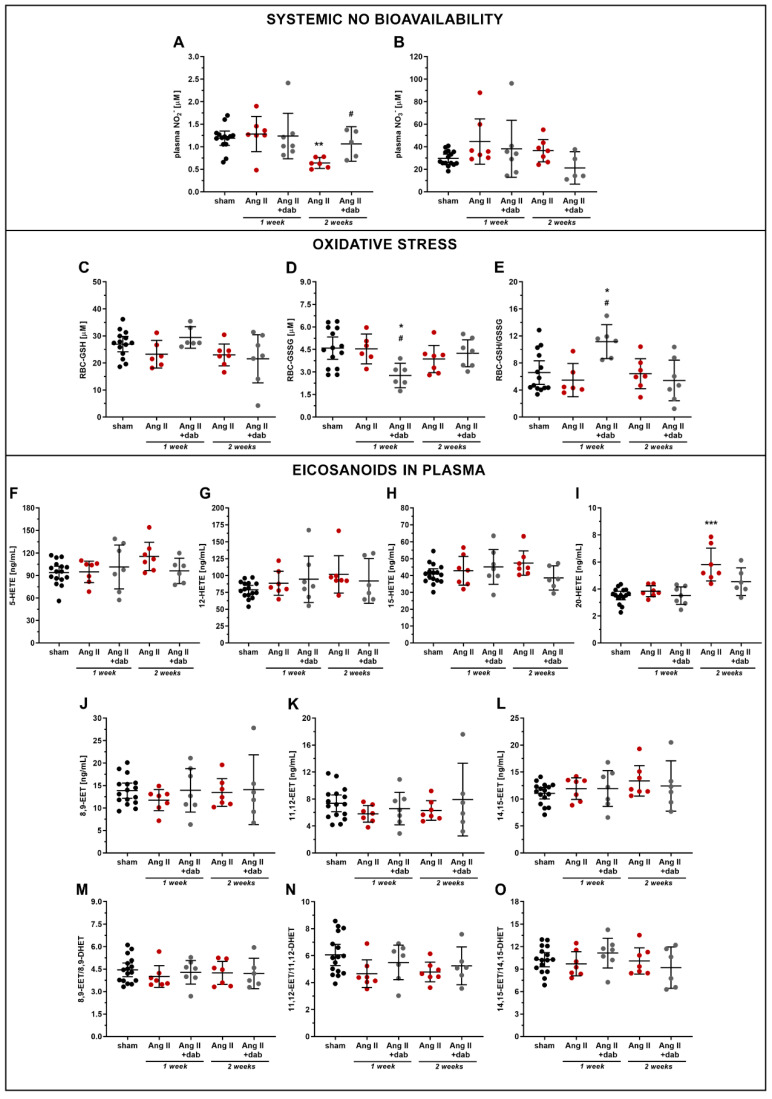
Effect of dabigatran on NO bioavailability, oxidative stress, and plasma eicosanoids in Ang II hypertensive mice. Concentration of plasma NO metabolites (**A**,**B**; *n* = 5–15) and oxidative stress markers including reduced glutathione GSH (**C**; *n* = 6–14), oxidised glutathione GSSG (**D**; *n* = 6–14), and their ratio (**E**; *n* = 6–14) measured in murine erythrocytes RBC as well as eicosanoid concentration in plasma (**F**–**O**; *n* = 7–14) were evaluated in mice subjected to *i.v.* continuous infusion of Ang II (144 µg/kg b.w. per day; 2 weeks) via catheters. Data are shown as means (–) ± 95% CI (I) and considered statistically significant at * *p* ≤ 0.05, ** *p* ≤ 0.01, and *** *p* ≤ 0.001, # *p* ≤ 0.05 using Tukey’s post hoc (**A**,**E**,**F**,**I**–**K**,**N**,**O**) and Kruskal–Wallis (**B**–**D**,**G**,**H**,**L**,**M**) statistical tests. * indicates statistical difference between sham mice and Ang II- or Ang II+ dab-treated animals, # indicates statistical difference between Ang II- and Ang II+ dab-treated mice.

In contrast to the one-week-long period of Ang II treatment, prolonged two-week administration of Ang II to mice resulted in a fall in plasma concentration of NO_2_^−^, whereas the NO_3_^−^ level remained unchanged (Figure 4A,B). Thrombin inhibition by dabigatran reversed the reduction in systemic NO bioavailability induced by prolonged Ang II administration as evidenced by increased plasma NO_2_^−^ concentration (Figure 4A). Moreover, dabigatran reversed Ang II-induced impairment of NO production in the aorta (Figure 3E) and was also associated with a notable reduction of positively stained aortic area for pro-inflammatory markers such as vWF and ICAM-1 (Figure S1). 

The impairment of systemic NO bioavailability induced by prolonged Ang II administration was not associated with a substantial change in oxidative stress measured in red blood cells (RBC) as GSH/GSSG balance (Figure 4C–E). Interestingly, prolonged Ang II administration resulted in an increase in 20-HETE plasma concentration, that was normalised by dabigatran treatment (Figure 4I). In turn, the plasma concentrations of 5-, 12-, and 15-HETE (Figure 4F–H) and 8,9-, 11,12-, and 14,15-EET (Figure 4J–L) as well as the EETs/DHETs ratio (Figure 4M–O) were not affected by Ang II nor dabigatran treatment in a prolonged model of Ang II-induced hypertension. However, plasma concentrations of some of eicosanoids were changed by a trend, including a slight increase in 5-HETE (93.94 and 115.50 ng/mL for sham and Ang II groups, respectively; *p* = 0.079) or 12-HETE (78.81 and 101.60 ng/mL for sham and Ang II groups, respectively; *p* = 0.066) (Figure 4F,G). 

## 3. Discussion

In the present work, we demonstrated that the direct inhibition of thrombin activity by dabigatran effectively prevented the development of Ang II-induced endothelial dysfunction and endothelial inflammation, however without affecting elevated blood pressure and vascular remodelling. Furthermore, prolonged (two-week-long), but not short-term (one-week-long) administration of Ang II was associated with a fall in systemic NO bioavailability and overproduction of 20-HETE, which were both reversed by dabigatran treatment. These results suggest that alterations in the systemic 20-HETE biosynthesis represent another time-dependent effect in Ang II hypertensive mice [17] and occur at the stage of significant impairment of systemic NO bioavailability, indicating that 20-HETE pathway could contribute to advanced phase of endothelial dysfunction associated with a systemic fall in NO bioavailability. 

Previous studies demonstrated that Ang II-induced endothelial dysfunction involves oxidative stress [18], endothelial dysfunction, and vascular inflammation [11,19], all of which were accompanied by vascular infiltration of leukocytes (e.g., T cells, myelomonocytic cells, macrophages) [19,20]. Additionally, Ang II-driven leukocyte invasion and adhesion to endothelium was shown to be dependent on factor XI (FXI) and subsequent thrombin activity via the interaction of platelet glycoprotein Ibα (GPIbα), which is a well-known thrombin receptor [21], and the integrin αMβ2 (CD11b/CD18 or Mac-1) localised on leukocytes [11,22]. Furthermore, the thrombin-dependent activation of protease-activated receptors (PARs) expressed on endothelial cells [13,14] could also contribute to endothelial dysfunction. Of note, the increased thrombin generation was also reported in the plasma of hypertensive patients [23] and other experimental models of hypertension [24,25]. Accordingly, in our work in the murine model of Ang II-induced hypertension, we extend the evidence supporting the involvement of thrombin in endothelial dysfunction and excluded the role of thrombin in vascular remodelling and hypertension [26,27].

It has been previously reported that activators or blockers of thrombin-activated PAR-1 evoke a decrease and increase in blood pressure in healthy animals, respectively [28], whereas the blockade of PAR-1 in renin-overexpressing mice reduces hypertension [29]. Moreover, PAR-1 receptors in the vasculature are known to contribute to endothelium-mediated vasodilatation and smooth muscle cell (SMC)-mediated vasoconstriction [30]. Nevertheless, our results in an Ang II model suggest that the sustained hypertension and vascular remodelling in mice treated with dabigatran are directly linked to effects of Ang II on the vasculature rather than to thrombin-dependent pathways. 

In contrast to the lack of effects of dabigatran on Ang II-related hypertension, vascular and cardiac remodelling (Figure S3), thrombin inhibition improved endothelial function as evidenced by the MRI-based in vivo measurements [31]. Indeed, dabigatran improved impaired FMD in the femoral artery and Ach-induced vasodilation in the thoracic aorta in Ang II-treated mice. The effects of dabigatran on endothelial function in the abdominal aorta were less significant, which was most likely due to the heterogeneous susceptibility of thoracic and abdominal parts of the mouse aorta to vascular insult [32].

Interestingly, one-week-long Ang II administration did not lower NO production by aorta ex vivo and systemic bioavailability of NO. On the other hand, prolonged two-week-long Ang II infusion resulted in a reduction of systemic NO bioavailability, which was associated with the increased biosynthesis of 20-HETE, which is a known endothelial mediator of vasoconstriction, eNOS uncoupling [33], and RAS activation [9,33,34]. Accordingly, an altered 20-HETE biosynthesis could contribute to the development of endothelial dysfunction in prolonged hypertensive models featuring a time-dependent systemic NO-deficiency, suggesting a reciprocal relation between NO and 20-HETE pathways in Ang II-induced hypertension [33,35]. Of note, this model was previously suggested to be 20-HETE-independent [33], in contrast to a dihydrotestosterone (DHT)-induced androgen-dependent model [36].

Apart from 20-HETE, the lipoxygenase (LOX)-derived HETEs including 5-, 12- and 15-HETE were also reported to influence endothelial function and hypertension [37,38]; however, their systemic and aorta biosynthesis (Figure S2) remained unchanged in response to Ang II treatment. The changes in HETE profile observed in our study suggest that eicosanoid-related mechanisms involved in Ang II-induced endothelial dysfunction and hypertension are rather governed by CYP450-derived (20-HETE) lipid mediators than LOX-dependent (5-, 12-, and 15-HETE) pathways.

It is also worth mentioning that the levels of CYP450-derived vasoprotective EETs [10] remained unchanged in mice with prolonged Ang II infusion. It is noteworthy that EETs (8,9-, 11,12- and 14,15-EET) are rapidly converted to their corresponding less-active diols (dihydroxyeicosatrienoic acids, DHETs) via soluble epoxide hydrolase (sEH) [39], and increased activity of this enzyme was observed under hypertensive condition [39,40,41]. Surprisingly, in our study, the systemic activity of sEH was not affected either by Ang II administration or by dabigatran as the EETs/DHETs ratio remained unchanged. On the other hand, the changes in renal sEH activity could be more pronounced as reported previously [39,40,41] than systemic response as assessed here based on the plasma EETs/DHETs measurements.

In the present work, we also demonstrated that the inhibition of thrombin activity by dabigatran markedly decreased the Ang II-associated endothelial inflammation. It was previously shown that the inhibition of thrombin activity by lepirudin decreases endothelial inflammation in Ang II-treated mice [11], and dabigatran attenuates the level of pro-inflammatory cytokines produced by stimulated peripheral blood mononuclear cells [42]. According to Kossmann et al., the anti-inflammatory effect of lepirudin results from the reduced infiltration of pro-inflammatory leukocytes to the vessel wall, and platelet GPIbα and FXI contribute to thrombin-dependent vascular inflammation [11], which stays in line with our observations (Figure S1D). Additionally, PAR-1 activation via thrombin triggers NF-κB-dependent pathways in endothelial cells and increases the expression of pro-adhesive, pro-inflammatory, and pro-coagulant molecules including VCAM-1, ICAM-1, and tissue factor (TF) [43].

Altogether, the reduction in Ang II-induced endothelial inflammation caused by dabigatran could have resulted from multiple mechanisms rather than only from the improvement of NO-dependent function and the normalisation of 20-HETE biosynthesis known to regulate endothelial inflammation [44,45].

In conclusion, thrombin activity inhibition by dabigatran effectively prevented the development of Ang II-induced endothelial dysfunction and endothelial inflammation, however without affecting hypertension and vascular remodelling. Furthermore, sustained hypertension induced by Ang II was associated with the reduction of systemic NO bioavailability and increased 20-HETE biosynthesis, which were reversed by dabigatran treatment. Our results underscore the close relationship between the NO- and 20-HETE-dependent pathways in Ang II hypertensive mice and suggest distinct mechanisms involved in Ang II-induced endothelial dysfunction and Ang II-induced hypertension being thrombin dependent and independent, respectively.

## 4. Materials and Methods

### 4.1. Animals

#### 4.1.1. Subcutaneous Ang II Administration via Micro-Osmotic Pumps

First, 12–14-week-old C57Bl/6J male mice were purchased from the Mossakowski Medical Research Centre of the Polish Academy of Sciences (Warszawa, Poland). All mice were kept under controlled environmental conditions with a light/dark cycle and fed with a standard chow diet and tap water ad libitum throughout the experiment. Mice were randomly divided into three of the following experimental groups: healthy mice after surgery without micro-osmotic pump implementation (sham, *n* = 10), and Ang II-treated mice with implemented micro-osmotic pumps without (Ang II, *n* = 10) or with dabigatran etexilate administration in chow (Ang II+dab, *n* = 10). The Ang II (A9525; Sigma Aldrich, St. Louis, MO, USA) solution was subcutaneously (*s.c.*) and continuously delivered via micro-osmotic pumps (0.21 µL/h; model 1002, Alzet, Cupertino, CA, USA) at a dose of 1 mg/kg b.w. per day, whereas the dose of dabigatran etexilate (BIBR-1048; Biorbyt, Cambridge, UK) was approximately 100 mg/kg b.w. per day. The implementation of micro-osmotic pumps was performed under isoflurane (Baxter Polska Sp. z o.o., Warszawa, Poland) anaesthesia using topical anaesthetics such as 2% lidocaine (Jelfa S.A., Jelenia Gora, Poland) and anti-septic 10% betadine (EGIS Polska Sp. z o.o., Warszawa, Poland). 

After one week of treatment, the endothelial function in vivo was assessed in each mouse by applying a magnetic resonance imaging (MRI) technique. On the next day, mice were euthanised using an intraperitoneal injection of ketamine (100 mg/kg b.w; Vetoquinol Biowet Sp. z o.o., Gorzow Wlkp., Poland) and xylazine (10 mg/kg b.w; Sigma Aldrich, St. Louis, MO, USA). Blood was drawn from the right ventricle using a syringe equipped with a plastic tip and centrifuged (664× *g*, 12 min, 4 °C). The collected plasma samples were stored at −80 °C for further analysis. After blood was taken, the aorta was isolated, cleaned up from fat and adherent tissue, and prepared for selected measurements. A part of aorta samples was fixed in 4% buffered formalin solution without cleaning the tissue.

All procedures carried out on animals were approved by the Second Local Ethical Committee on Animal Testing in the Institute of Pharmacology, Polish Academy of Sciences (Krakow, Poland; permit no. 319/2018) and performed according to the guidelines from Directive 2010/63/EU of the European Parliament on the protection of animals used for scientific purposes.

Short-term subcutaneous administration of Ang II to mice was conducted to observe early changes associated with Ang-II induced hypertension and endothelial dysfunction.

#### 4.1.2. Intravenous Ang II Administration and Blood Pressure Measurements

First, 12–14-week-old C57Bl/6J male mice weighting 25–30 g were obtained from Taconic (Lille Skensved, Denmark). After delivery, the animals were housed under controlled temperature and humidity conditions in a room with a 12-hour light/dark cycle. Mice were fed with a standard chow diet and tap water ad libitum throughout the experiment.

Prior to surgery, mice were anaesthetised using 100 mg/kg b.w. ketamine (MSD, Boxmeer, Netherlands) and 10 mg/kg b.w. xylazine (KVP Pharma+Veterinär Produkte GmbH, Kiel, Germany); then, they were injected with 500 µL of saline. For simultaneous intravenous administration of Ang II and blood pressure measurements, micro-renathane tip-based catheters connected to polyethylene tubing were placed into a femoral vein and a femoral artery [46]. Next, the catheters were pulled subcutaneously, exteriorised via skin on the neck, filled with heparin solution (LEO Pharma A/S, Ballerup, Denmark) (100 IU/mL in isotonic glucose; Amgros I/S, Copenhagen, Denmark), and connected to a swivel system (Instech Laboratories, PA, USA), which enabled free movement of the individually housed animals. Mice received a subcutaneous injection of buprenorphinum (Temgesic; Indivior UK Ltd, Slough, UK) at a dose of 3.75 mg/kg b.w. to relieve post-operative pain, and additional twice intravenous injections at an 8-hour interval via the pump-system. After a 5-day recovery period, the experimental procedures were started. Animals were randomly divided into the following experimental groups: Ang II-induced hypertensive mice (Ang II, n = 9) and Ang II-induced hypertensive mice treated with dabigatran etexilate in chow (Ang II+dab, *n* = 9). The solution of Ang II (A9525; Sigma Aldrich, St. Louis, MO, USA) was continuously infused by syringe pumps (10 µL/h) via a femoral vein catheter at a dose of 144 µg/kg b.w. per day, whereas the dose of dabigatran etexilate (BIBR-1048; Biorbyt, Cambridge, UK) was approximately 100 mg/kg b.w. per day. Due to health conditions, three out of eighteen operated mice did not survive the whole experimental period.

The catheter placed into the femoral artery was connected to a pressure transducer (Fӧhr Medical Instruments, Hessen, Germany), and the mean arterial pressure (MAP) and heart rate (HR) data were recorded continuously throughout the entire experiment using LabView software (National Instruments, Austin, TX, USA). After a period of collecting baseline MAP and HR, the infusion of Ang II and treatment with dabigatran etexilate commenced.

Apart from blood pressure data collection, the arterial catheter allowed for multiple blood collection from mice (200 µL of blood each time, three times in total) without anaesthesia including the following time points: 5 days after surgery (sham), 7 days after Ang II or Ang II+dabigatran treatment (Ang II and Ang II+dab 1 week), and 14 days after Ang II or Ang II+dabigatran administration (Ang II and Ang II+dab 2 weeks). After every blood collection via a catheter, the body fluid volume was filled with a saline and heparin solution at a concentration of 100 IU/mL in isotonic glucose (200 µL in total). After blood centrifugation (664× *g*, 12 min, 4 °C), the plasma was collected and kept at -80 °C for further analysis. At the end of the experiment, mice were euthanised after isoflurane (Baxter A/S, Allerød, Denmark) inhalation anaesthesia using a cardiac puncture to collect blood samples. Subsequently, the heart was removed. Next, the aorta was isolated, cleaned up from fat and adherent tissue, and prepared for selected measurements. The thoracic aorta rings of mice (3–4 mm long) were also immersed in an optimal cutting temperature (OCT) compound and immediately frozen at −80 °C. 

All animal experiments were performed complying with Danish Law under the animal experimental permit 2015-15-0201-00479 issued by the Dyreforsøgstilsynet animal committee (Glostrup, Denmark) and according to the guidelines from Directive 2010/63/EU of the European Parliament on the protection of animals used for scientific purposes.

Prolonged intravenous administration of Ang II to mice was conducted to observe advanced changes associated with Ang II-induced hypertension and endothelial dysfunction.

Due to the given numerous end-point measurements performed in the presented study, the planned groups of animals were divided into subgroups. The exact number of animals used in the particular measurements was indicted in the figure legends.

### 4.2. Measurements of Thrombin Activity (CAT) and Dabigatran Concentration in Plasma 

The effect of dabigatran on thrombin activity was measured in murine plasma using thrombin generation assay according to Tchaikovsky et al. [47] with major modifications. At the beginning, citrated mouse plasma was mixed with fluorogenic substrate (Z-Gly-Gly-Arg-AMC; Diagnostica Stago, Asnières sur Seine Cedex, France) solution and subsequently pipetted into the wells of a detection plate. Then, thrombin generation in plasma was initiated by the addition of a trigger solution containing tissue factor (TF), phospholipids (PL), and CaCl_2_ (Merck, Darmstadt, Germany). As a result, 60 µL of the prepared mixture in wells consisted of 12 µL of plasma, 9 µL of substrate solution, and 39 µL of trigger solution at a final concentration of 20% plasma, 1.0 pM TF, 16.25 mM CaCl_2_, 4 µM PL, and 0.43 mM ZGGR-AMC. Each plasma sample was calibrated by replacing the trigger solution with a solution containing α2-macroglobulin–thrombin complex (α2M-T, at a final concentration corresponding to 44 nM thrombin activity). Measurements were performed at 37 °C, and each sample was tested in duplicate. Fluorescent signals were recorded using a Tecan Spark 10M microplate reader (Männedorf, Switzerland) and transformed into thrombin concentration as described previously [48]. The effect of dabigatran was evaluated based on a lag-time parameter, representing time to start of thrombin generation. The reagents such as TF, PL and α2M-T were provided as a gift by Synapse Research Institute (Maastricht, Netherlands).

Dabigatran etexilate is an oral prodrug that is hydrolysed to the direct thrombin inhibitor dabigatran. The concentration of dabigatran was assessed in mouse plasma samples using a UPLC-MS/MS technique. A plasma volume of 50 µL was spiked with 5 µL of internal standard (dabigatran-^13^C_6_; TRC, Toronto, Canada) at a concentration of 1 µg/mL. After gently shaking, 150 µL of 0.1 M HCl in MeOH (WITKO Group, Lodz, Poland) was added, mixed for 10 min, and chilled at 4 °C for the next 10 min. The supernatant collected after sample centrifugation (16,600× *g*, 15 min, 4 °C) was directly injected into an UltiMate 3000 UPLC system (Thermo Fisher Scientific, Waltham, MA, USA) combined with a TSQ Quantum Ultra triple quadrupole mass spectrometer (Thermo Fisher Scientific, Waltham, MA, USA). The chromatographic analysis was conducted using an Acquity UPLC BEH C18 (3.0 × 100 mm^2^, 1.7 μm; Waters, Milford, MD, USA) analytical column and applying 0.1% formic acid (FA; Thermo Fisher Scientific, Waltham, MA, USA) in ACN (Sigma Aldrich, St. Louis, MO, USA) (A) and 0.1% FA in H_2_O (B) as mobile phases delivered in the following gradient elution program: 95% B hold for 1 min, 95–5% B for 3 min, 5–95% B for 0.5 min, and 95% B for 2.5 min for column equilibration. The mass spectrometric detection was conducted in an electrospray positive ionisation mode, and selected ion transitions were used for quantification: 472.4→172.0 (CE = 39 V) and 478.3→172.1 (CE = 39 V) for dabigatran and dabigatran-^13^C_6_, respectively. The mass spectrometry operating parameters were as follows: spray voltage = 5000 V, vaporiser temperature = 300 °C, auxiliary gas = 25, and sheath gas = 30.

### 4.3. Assessment of In Vivo Endothelial Function by Magnetic Resonance Imaging (MRI)

The in vivo approach for endothelial function assessment using a magnetic resonance imaging (MRI) technique was developed, successfully applied, and previously described by Bar et al. [31]. MRI experiments on mice were conducted using a 9.4T scanner (BioSpec 94/20 USR, Bruker, BioSpin GmbH, Germany) under isoflurane anaesthesia (Baxter Polska Sp. z o.o., Warszawa, Poland; 1.5 vol%) in oxygen and air (1:2) mixture and constant body temperature maintained at 37 °C using a circulating warm water system. The heart activity, respiration, and body temperature were monitored by applying a Monitoring and Gating System (SA Instruments Inc., Stony Brook, NY, USA). The endothelial function was evaluated in response to reactive hyperaemia applying a flow-mediated vasodilation (FMD) method as well as in response to acetylcholine (Ach; Sigma Aldrich, St. Louis, MO, USA) administration. 

The home-made equipment for FMD measurements in mice allowed for a short-term occlusion (5 min) of a mouse femoral artery (FA) and examination of volume changes of the FA in response to occluder release and increased blood flow. Three-dimensional (3D) images of FA were recorded on the coronal view of the mice (on their left hind limb). 

The vessel response to acetylcholine administration (Ach, *i.p*, 16.6 mg/kg b.w) was assessed in the thoracic (ThA) and abdominal (AbA) aorta. Vasomotor response was evaluated by comparing two time-resolved 3D images of the vessels prior to and 25 min after Ach injection. Three-dimensional (3D) images of the ThA and AbA were acquired on the sagittal view of the mice, approximately 5 mm under the heart.

All images were registered using the cine IntraGateTM FLASH 3D sequence and reconstructed with the IntraGate 1.2.b.2 macro (Bruker, Bremen, Germany). End-diastolic volumes of vessels were analysed using ImageJ software 1.46r (NIH, Bethesda, MD, USA) and scripts written in Matlab (MathWorks, Natick, MA, USA). The detailed imaging parameters were described in our previous work [31].

### 4.4. Measurements of Endothelium-Derived NO Production in Aorta by EPR

Ex vivo endothelial function was assessed based on the release of NO from isolated mouse aorta applying an electron paramagnetic resonance (EPR) spin trapping approach. According to a previously described protocol [49], the cleaned mouse thoracic aorta was opened longitudinally and pre-incubated in Krebs–Hepes buffer in the presence of L-NIL (Cayman Chemical, Ann Arbor, MI, USA) for 30 min; then, a freshly prepared Fe(DETC)_2_ spin trap and calcium ionophore (Sigma Aldrich, St. Louis, MO, USA) were added obtaining the final concentrations of 285 μM and 1 μM, respectively. After 90 min of incubation, each aorta was drained on a Kimwipe, weighted, placed into the middle of a column of Krebs–Hepes buffer in a dedicated vessel, and immediately frozen in liquid nitrogen. Samples were stored at −80 °C until NO-Fe(DETC)_2_ adducts were measured using an X-band EPR spectrometer EMX Plus (Bruker, Bremen, Germany). NO release was expressed as the EPR amplitude of the second hyperfine line of the acquired NO-Fe(DETC)_2_ spectra in arbitrary units and normalised to the aorta weight.

### 4.5. Measurements of NO Metabolites in Plasma

The concentrations of NO metabolites including nitrite (NO_2_^−^) and nitrate (NO_3_^−^) in plasma samples were determined using an HPLC-based system ENO-20 NOx Analyser (Eicom, Kyoto, Japan) following a previously published protocol [50]. Briefly, after plasma sample precipitation with MeOH (1:1, *v*/*v*) and centrifugation (10,000× *g*, 10 min, 4 °C), 10 µL of supernatant was directly injected into the system. Next, NO_3_^−^ was reduced on a cadmium-cooper NO-RED column to NO_2_^−^ and subsequently mixed with Griess reagent. The absorbance of generated diazo derivatives was measured at λ = 540 nm. All essential materials for NO metabolite measurements, including chromatographic columns as well as carrier and reactor powders, were obtained from Eicom (Kyoto, Japan).

### 4.6. Analysis of Endothelial Phenotype by Immunohistochemistry (IHC)

The aorta expression of VEGF-A, HIF-1α, SDF-1α, eNOS, and vWF was evaluated in paraffin-embedded aortic fragments preserved from mice subjected to *s.c.* administration of Ang II, while OCT-embedded aortic rings were obtained from mice subjected to *i.v.* administration of Ang II and were used for vWF, ICAM-1, and VCAM-1 immunostaining.

Paraffin-embedded aortic fragments were cut into 5 µm-thick cross-sections using a Sakura Accu-Cut 200 rotary microtome (Sakura, Osaka, Japan), collected on microscopic slides (Menzel Glaser SuperFrost, Braunschweig, Germany), deparaffinised, and treated with a citrate buffer-based heat-induced antigen retrieval (HIER) protocol. In case of OCT-embedded aortic fragments, samples were cut into 10 µm-thick cross-sections using a Leica CM1860 cryostat (Leica Biosystems, Wetzlar, Germany), collected on polylisine-covered microscopic slides (Menzel Glaser SuperFrost), fixed with 4% paraformaldehyde, washed in distilled water, dried, and stored at room temperature. All aorta cross-sections were pre-incubated for 30 min with blocking buffer solution, containing 2% dry milk (Gostyn, Poland) and 5% normal goat serum (Jackson ImmunoResearch, Cambridgeshire, UK). Slides intended for immunostaining with mouse antibodies were additionally incubated with MOM blocking reagent (Vector, Burlingame, CA, USA) to reduce the unspecific background from endogenous antibodies. The following primary antibodies were applied for overnight incubation: anti-VEGF-A (ab51745; Abcam, Cambridge, UK), anti-HIF-1α (H1alpha67; Abcam, Cambridge, UK), SDF-1α (orb251479; Biorbyt, Cambridge, UK), anti-eNOS (610296; BD Biosciences, Franklin Lakes, NJ, USA), anti-vWF (ab6994; Abcam, Cambridge, UK), anti-ICAM-1 (14-0542-82; Thermo Fisher Scientific, Waltham, MA, USA), and anti-VCAM-1 (MA5-11447; Thermo Fisher Scientific, Waltham, MA, USA). 

The immunostained slides subjected to VEGF-A, HIF-1α, SDF-1α, and eNOS histochemical analysis were incubated with biotin-conjugated goat-anti-mouse or goat-anti-rabbit secondary antibody (Jackson ImmunoResearch, Cambridgeshire, UK) followed by incubation with VECTASTAIN Elite ABC-HRP Kit (PK-6100; Vector Laboratories, Burlingame, CA, USA) and diaminobenzidine (Sigma Aldrich, St. Louis, MO, USA) to obtain the colour reaction. Subsequently, the cross-sections were photographed (100× magnification) using a BX51 microscope (Olympus, Tokyo, Japan). Before analysis in the immunostained pictures, non-adipose tissue fragments (aorta wall, muscles, lymph nodes) were manually excised. Image segmentation was performed automatically using Ilastik (developed by the Ilastik team, with partial financial support of the Heidelberg Collaboratory for Image Processing, HHMI Janelia Farm Research Campus and CellNetworks Excellence Cluster). The algorithm classifies pixels based on identical criteria of image properties (colour, edge, and texture) defined by the specialist of histology. The immunopositive pixels were quantitatively determined using ImageJ software 1.46r. All results were normalised for circuit of the aorta lumen.

The immunofluorescence stained slides subjected to vWF, ICAM-1, and V-CAM analyses were treated with secondary antibodies: Cy3-conjugated goat-anti-mouse, Cy3-conjugated goat-anti-rabbit, and Alexa Fluor 488-conjugated goat-anti-rat (Jackson ImmunoResearch, Cambridgeshire, UK). For nuclei counterstaining, Hoechst 33,258 solution (Sigma Aldrich, St. Louis, MO, USA) was applied. Immunostained sections were photographed using an AxioObserver.D1 inverted fluorescent microscope connected to an AxioCam HRm monochromatic camera (Carl Zeiss, Oberkochen, Germany), stored as tiff files, and analysed using Zeiss ZEN software. The results were normalised to elastin area. 

### 4.7. Assessment of Aorta Vascular Wall Thickness by Histology

For the determination of aorta wall, intima-media, and adventitia thickness, 4% formalin-fixed thoracic aorta rings were embedded in paraffin, and 5 μm-thick serial sections of the aorta were collected. Next, the staining method with OMSB was applied on every tenth section (50 μm interval between each section) as described previously [51]. The thicknesses of aorta wall, intima-media, and adventitia were manually evaluated at 12 measurement points, including three different slices of the aorta cross-section from one mouse using Olympus VS-ASW Virtual Slide System processing software. Samples were photographed at 400× *g* magnification with an Olympus BX51 light microscope (Olympus Corporation, Tokyo, Japan).

### 4.8. Measurements of Eicosanoid Production in Full Blood

Eicosanoid generation in full blood ex vivo was accomplished using a specially designed Xyzyk apparatus (Xyzyk Company, Krakow, Poland) [52]. Freshly collected citrated blood samples were diluted 5-fold with saline solution and incubated at 37 °C for 60 min, with constant stirring by microdipol to activate eicosanoid release (1500 rpm; rotation direction changed every 3 s). After 60 min of stirring, samples of blood were transferred into 500 µM acetylsalicylic acid solution in Eppendorf tubes, incubated for 2 min, and then centrifuged (664× *g*, 12 min, 4 °C). After centrifugation, plasma samples were stored at −80 °C for eicosanoid quantification using a UPLC-MS/MS technique.

### 4.9. Measurements of Eicosanoid Production in Aorta

A cleaned abdominal part of mouse aorta was conditioned for 15 min in Krebs–Hepes buffer at 37 °C. After pre-incubation, the tissue was transferred to a fresh Krebs–Hepes buffer (500 µL) and further incubated for 60 min with 1 µM arachidonic acid (AA, 10006607; Cayman Chemical, Ann Arbor, MI, USA). Next, the collected buffer was frozen and kept at −80 °C for eicosanoid quantification via a UPLC-MS/MS technique, whereas the aorta was dried using Kimwipe tissue and frozen at −80 °C for further protein content analysis. The concentration of eicosanoids produced by aorta was normalised to mg of protein determined in aorta homogenates.

### 4.10. UPLC-MS/MS Eicosanoid Analysis 

Selected eicosanoids (5-, 12-, 15-, and 20-HETE, 8,9-, 11,12-, and 14,15-EET, 8,9-, 11,12-, and 14,15-DHET) were quantified in plasma, Xyzyk-derived plasma, and Krebs–Hepes buffer collected after aorta incubation using a UPLC-MS/MS technique with the application of an already published methodology [53]. In short, each sample was spiked with a mixture of internal standards and gently mixed. Plasma samples were precipitated using MeOH (WITKO Group, Lodz, Poland). After vigorous shaking and centrifugation, the supernatant was transferred to a fresh tube, and 10% FA (Thermo Fisher Scientific, Waltham, MA, USA) was added. Next, samples were extracted twice using dichloromethane (DCM; Merck, Darmstadt, Germany) and mixed after each addition of organic solvent. Then, MiliQ water was added, and samples were thoroughly vortexed followed by centrifugation. In the next step, the bottom layer was collected and evaporated to dryness under a nitrogen stream. The dry residue was dissolved in 1.25 M NaOH (Sigma Aldrich, St. Louis, MO, USA), incubated at 90 °C (20 min), and mixed every 5 min. After the hydrolysis process, samples were chilled in an ice bath, and 10% FA was added. Then, samples were extracted using DCM and mixed. After centrifugation, the organic bottom layer was transferred to a fresh tube and evaporated to dryness under a nitrogen stream. 

In the case of incubation buffer samples, eicosanoids were extracted using acidified ethyl acetate (Merck, Darmstadt, Germany), and after centrifugation, the upper organic layer was transferred to a fresh tube and dried under a nitrogen stream. 

The sample dry residue was dissolved in EtOH (J.T Baker, Phillipsburg, NJ, USA) and samples were injected into the UPLC-MS system consisted of a UFLC Nexera liquid chromatograph (Shimadzu, Kyoto, Japan) coupled to a triple quadrupole mass spectrometer QTrap 5500 (Sciex, Framingham, MD, USA). The separation of analytes was performed on an Acquity UPLC BEH C18 (3.0 × 100 mm^2^, 1.7 μm; Waters, Milford, MD, USA) under gradient elution mode applying 0.1% FA in ACN and 0.1% FA in H_2_O (*v*/*v*) as mobile phases. The mass spectrometry detection parameters of analytes and used internal standards were thoroughly listed in our previous work [53].

### 4.11. Measurements of GSH and GSSG in Red Blood Cells

Measurements of GSH and GSSG were performed by capillary electrophoresis according to a protocol described by Hempe et al. [54]. Briefly, 200 µL of a mixture of 10 mM KCN (Sigma Aldrich, St. Louis, MO, USA) and 5 mM EDTA (Sigma Aldrich, St. Louis, MO, USA) prepared in deionised water (haemolysing reagent) was added to 50 µL of erythrocytes. Then, 100 µL of haemolysate was precipitated with 100 µL of 5% metaphosphoric acid (MPA; Sigma Aldrich, St. Louis, MO, USA). After centrifugation (10,000× *g*, 10 min, 4 °C), the MPA extracts were diluted with deionised water (1:4, *v*/*v*) and injected onto a CE system comprising a P/ACE MDQ capillary electrophoresis machine (Beckman Coulter, Fullerton, CA, USA) equipped with a PDA detector. Separation of the analytes took place in an uncoated fused-silica capillary (60.2 cm total length, 50 cm effective length, 50 μm i.d., and 375 μm o.d.) thermostated at 25 °C with a constant voltage of 25 kV (≈6.5 µA). A mixture of BisTRIS (75 mM; Sigma Aldrich, St. Louis, MO, USA) and boric acid (25 mM; J.T Baker, Phillipsburg, NJ, USA) adjusted to pH 7.8 by the addition of 1 M NaOH (Sigma Aldrich, St. Louis, MO, USA) was applied as a background electrolyte (BGE). Studied samples were introduced to the capillary via hydrodynamic injection for 20 s at 3.5 kPa, followed by an injection of ultrapure H_2_O for 2 s at 3.5 kPa. Between analytical runs, the capillary was rinsed with 1 M NaOH, deionised water, and BGE (138 kPa; 2 min each). The absorbance of GSH and GSSG was detected at λ = 200 nm.

### 4.12. Total Protein Determination in Aorta Homogenates

The concentration of total proteins in aorta homogenates was measured using a Pierce™ BCA Protein Assay Kit (23225; Thermo Fisher Scientific, Waltham, MA, USA) following the manufacturer’s instructions. Aorta samples were homogenised automatically using Precellys Evolution combined with a Cryolys cooling unit (Bertin, Montigny-le-Bretonneux, France). After centrifugation (10,000× *g*, 10 min, 4 °C), the supernatant was analysed for total protein concentration.

### 4.13. Statistics

Data were presented as the mean ± 95% CI and plotted using GraphPad Prism 8.2.1 software (GraphPad Software Inc., La Jolla, CA, USA). All quantitative results were statistically analysed applying the adequate parametric tests (T-test or ANOVA with Tukey’s post hoc test) or non-parametric calculations (U-Mann–Whitney and Kruskal–Wallis ANOVA tests) available in Statistica 13.1 (Statistica, Tulsa, OK, USA). Results were considered statistically significant at *p*-values equal to or below 0.05.

## Figures and Tables

**Table 1:** Effect of dabigatran on eicosanoid production in full blood ex vivo.

Eicosanoid Production in Full Blood Ex Vivo *n* = 9–10	Sham	Ang II	Ang II + Dab
5-HETE (ng/mL) ^A^	14.67 (13.05, 16.28)	18.22 (15.16, 21.28)	18.19 (15.41, 20.98)
12-HETE (ng/mL) ^B^	496.06 (372.10, 620.01)	553.11 (254.59, 851.62)	526.83 (228.08, 825.59)
15-HETE (ng/mL) ^B^	13.49 (11.59, 15.38)	16.30 (12.68, 19.92)	16.74 (13.81, 19.67)
20-HETE (ng/mL) ^A^	1.26 (0.99, 1.53)	1.31 (1.16, 1.46)	1.25 (0.95, 1.55)
8,9-EET (ng/mL) ^A^	4.65 (4.13, 5.17)	5.71 (4.78, 6.65)	5.05 (4.21, 5.88)
11,12-EET (ng/mL) ^B^	3.30 (2.85, 3.76)	4.08 (3.57, 4.58)	3.67 (3.00, 4.34)
14,15-EET (ng/mL) ^B^	2.92 (2.45, 3.39)	3.74 (3.10, 4.39)	3.14 (2.47, 3.81)
8,9-EET/8,9-DHET ^B^	4.09 (3.77, 4.42)	4.17 (3.53, 4.81)	3.76 (3.27, 4.25)
11,12-EET/11,12-DHET ^A^	4.21 (3.89, 4.52)	4.91 (4.23, 5.59)	4.54 (4.07, 5.01)
14,15-EET/14,15-DHET ^A^	4.33 (3.77, 4.89)	5.20 (4.75, 5.65)	4.30 (3.53, 5.08)

Eicosanoid production in full blood ex vivo after mechanical stimulation applying the XYZYK system was assessed in mice subjected to *s.c.* administration of Ang II (1 mg/kg b.w. per day; 1 week) via micro-osmotic pumps. Data are shown as mean ± 95% CI, and the statistical analysis was performed using Tukey’s post hoc ^A^ or Kruskal–Wallis ^B^ statistical tests. DHETs-dihydroxyeicosatrienoic acids.

## Data Availability

The data presented in this study are available on reasonable request from the corresponding author.

## Supplementary Materials

The following are available online at https://www.mdpi.com/article/10.3390/ijms22168664/s1.
