# Supplementary material for: Thrombin Inhibition Prevents Endothelial Dysfunction and Reverses 20-HETE Overproduction without Affecting Blood Pressure in Angiotensin II-Induced Hypertension in Mice
Source: Int J Mol Sci. 2021 Aug 12;22(16):8664. doi: 10.3390/ijms22168664 (PMC8395447; doi:10.3390/ijms22168664)
Supplement: Supplementary file 1 [file ijms-22-08664-s001.zip › ijms-1333391-supplementary.pdf]

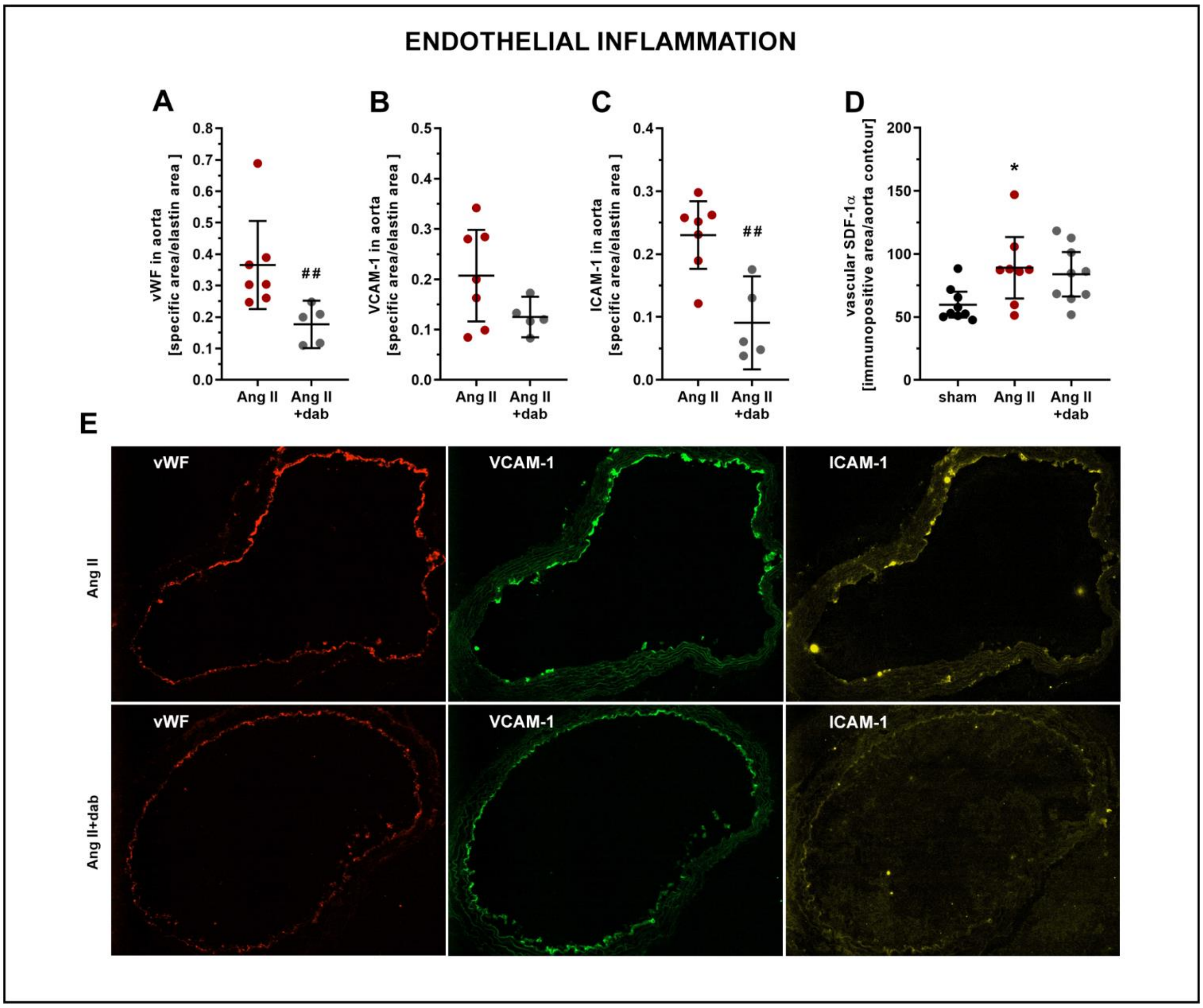

Figure S1 Effect of dabigatran on endothelial inflammation in the aorta of Ang II hypertensive mice. The aorta area positively stained for pro-inflammatory markers normalised to the elastin area including vWF (**A**; *n = 5–7*), VCAM-1 (**B**; *n* = 5–7) and ICAM-1 (**C**; *n* = 5–7) was evaluated in mice subjected to *i.v* continuous infusion of Ang II (144 µg/kg b.w per day; 2 weeks) via catheters. The aorta area positively stained for modulator of monocyte migration SDF-1α (**D**; *n* = 8) was assessed in mice subjected to *s.c* administration of Ang II (1 mg/kg b.w per day; 1 week) via micro-osmotic pumps. Representative images of the OCT-embedded thoracic aorta cross-sections after immunohistochemistry (IHC) staining (**E**). Data are shown as means (–) ± 95% CI (I) and considered statistically significant at $^{\#\#}p \leq 0.01$ using t-test (A, C), U-Mann-Whitney (B) and Tukey's post hoc (D) statistical tests. # indicates statistical difference between Ang II- and Ang II+dab-treated mice.

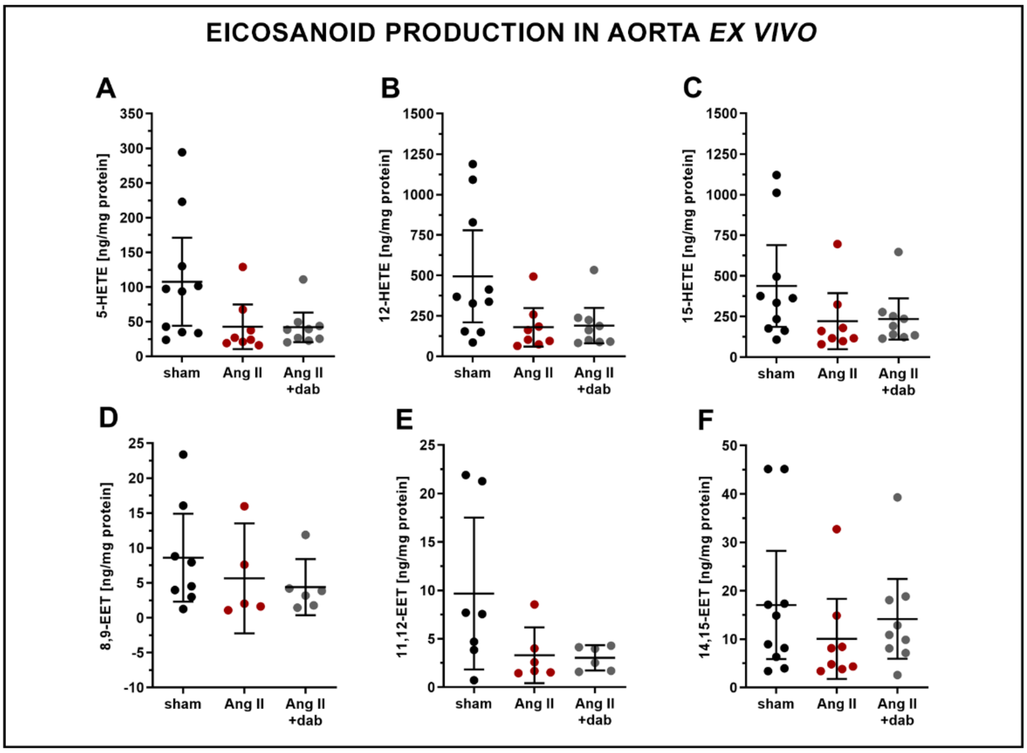

Figure S2 Effect of dabigatran on eicosanoid production in the aorta of Ang II hypertensive mice. Eicosanoid production in the abdominal aorta assessed in incubation buffer after the addition of arachidonic acid (AA, 1 μM) was measured in mice subjected to *s.c* administration of Ang II (1 mg/kg b.w per day; 1 week) via micro-osmotic pumps (**A-F**, *n* = 5–10). Concentration of 20-HETE and DHETs was not detectable in majority of studied samples. Data are shown as means (–) ± 95% CI (I).

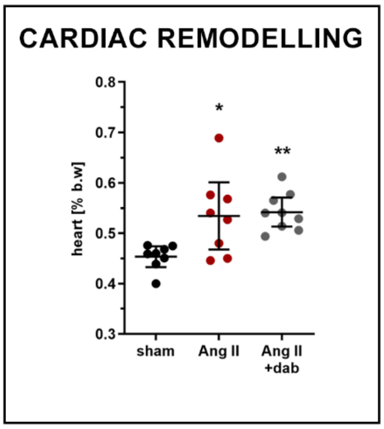

Figure S3 Effect of dabigatran on cardiac remodelling in Ang II hypertensive mice. Heart weight normalised to body weight (n = 8–9) was assessed in mice subjected to *s.c* administration of Ang II (1 mg/kg b.w per day; 1 week) via micro-osmotic pumps. Data are shown as means (–) ± 95% CI (I) and considered statistically significant at $^{*}p \leq 0.05$, $^{**}p \leq 0.01$ using Kruskal-Wallis statistical tests. * indicates statistical difference between sham mice and Ang II- or Ang II+dab-treated animals.
